# Characteristics of Patients Receiving Novel Muscular Dystrophy Drugs in Trials vs Routine Care

**DOI:** 10.1001/jamanetworkopen.2023.53094

**Published:** 2024-01-24

**Authors:** Dongzhe Hong, Jerry Avorn, Richard Wyss, Aaron S. Kesselheim

**Affiliations:** 1 Program on Regulation, Therapeutics, and Law (PORTAL), Division of Pharmacoepidemiology and Pharmacoeconomics, Department of Medicine, Brigham and Women’s Hospital/Harvard Medical School, Boston, Massachusetts; 2 Division of Pharmacoepidemiology and Pharmacoeconomics, Department of Medicine, Brigham and Women’s Hospital/Harvard Medical School, Boston, Massachusetts

## Abstract

**Importance:**

The US Food and Drug Administration approved eteplirsen for Duchenne muscular dystrophy (DMD) in 2016 based on a controversial pivotal study that demonstrated a limited effect on the surrogate measure of dystrophin production. Other DMD treatments in the same class followed.

**Objective:**

To assess how patients receiving novel DMD treatments in postapproval clinical settings compare with patients in the clinical trials.

**Design, Setting, and Participants:**

This cross-sectional study collected data on patients who initiated 1 of 4 novel DMD treatments (eteplirsen, golodirsen, viltolarsen, and casimersen) using national claims databases of commercially insured (Merative MarketScan and Optum’s Clinformatics Data Mart Database [CDM]) and Medicaid patients between September 19, 2016, and March 31, 2022. Patients were followed for 1 year after the date of first use of any novel DMD treatment. In addition, patients in pivotal DMD drug trials were identified for comparison.

**Exposures:**

Age, sex, race and ethnicity, region, and DMD stage of patients receiving novel DMD treatment.

**Main Outcome and Measures:**

The main outcome was health care costs and drug discontinuation as measured using descriptive statistics.

**Results:**

A total of 223 routine care patients initiating novel DMD drugs (58 in MarketScan, 35 in CDM, and 130 in Medicaid) were identified. Among the 106 patients in the pivotal trials, the mean (SD) age was 8.5 (2.0) years (range, 4.0-13.0 years), which was younger than the mean age of patients in routine care (MarketScan: 13.7 [7.0] years [range, 1.8-33.3 years; *P* < .001]; CDM: 11.9 [5.7] years [range, 0.6-23.6 years; *P* < .001]; Medicaid: 13.4 [6.5] years [range, 1.8-46.1 years; *P* < .001]). The proportion of female patients identified in postapproval clinical settings was 2.9% (n = 1) in CDM (vs 34 male patients [97.1%]) and 1.5% (n = 2) in Medicaid (vs 128 male patients [98.5%]), which was not different from the pivotal trials. While nearly all patients in the pivotal trials had DMD disease stage 1 or 2 when initiating the DMD treatments (103 [97.2%]), in the postapproval clinical setting, slightly more than one-third of patients were in disease stage 3 or 4 (MarketScan, 17 [36.2%; *P* < .001]; CDM, 13 [41.9%; *P* < .001]; Medicaid, 54 [47.0%; *P* < .001]). The payer’s cost for novel DMD treatments varied across the databases, with a mean (SD) of $634 764 ($607 101) in MarketScan, $482 749 ($582 350) in CDM, and $384 023 ($1 165 730) in Medicaid. Approximately one-third of routine care patients discontinued the treatments after approximately 7 months (mean [SD], 6.1 [4.4], 6.9 [3.9], and 7.2 [4.3] months in MarketScan, CDM, and Medicaid, respectively).

**Conclusions and Relevance:**

These findings raise questions about the translation of DMD drug trial findings to routine care settings, with patients in routine care discontinuing the treatment within 1 year and payers incurring substantial expenses for these medications. More data are needed on whether these high costs are accompanied by corresponding clinical benefits.

## Key Points

**Question:**

What are the differences in demographic and disease characteristics of patients receiving novel Duchenne muscular dystrophy (DMD) drugs in clinical trials compared with routine care settings?

**Findings:**

In this cross-sectional study of 223 routine care patients and 106 patients in pivotal trials, patients prescribed novel DMD treatments during routine care were in later stages of disease and older than those in clinical trials. Routine care patients discontinued treatment after approximately 7 months, and payers incurred substantial expenses for these medications.

**Meaning:**

These findings indicate that novel drugs to treat DMD are expensive and often discontinued in routine care settings, warranting further study into whether high drug costs are accompanied by corresponding clinical benefits.

## Teaser

This cross-sectional study assesses the characteristics of patients with Duchenne muscular dystrophy receiving novel treatment in routine care compared with patients in clinical trials.

## Introduction

Duchenne muscular dystrophy (DMD) is a genetic disorder caused by almost total lack of the muscle dystrophin protein and characterized by progressive muscle weakness and wasting. The disease typically manifests in early childhood and leads to mobility impairment, respiratory complications, cardiac involvement, and ultimately death, usually by the third decade.^1^ Corticosteroids, assistive devices, and cardiac and respiratory support as the disease progresses have been available to manage symptoms and preserve patients’ quality of life.^2,3,4^ Since 2016, exon-skipping therapy has emerged as a new class of treatment for DMD.

The US Food and Drug Administration (FDA) granted accelerated approval in 2016 for eteplirsen (Exondys 51; Sarepta Therapeutics), an antisense oligonucleotide therapy designed to increase dystrophin production by skipping specific exons in a subset of patients with DMD.^5^ Eteplirsen’s approval was met with controversy related to the pivotal trial, which enrolled 12 patients and demonstrated only very small increases in the surrogate measure end point of dystrophin levels, suggesting that it may not have any real clinical benefit. Immediately after approval, the manufacturer set a price of approximately $300 000 per year for patients with typical weight, although the price of the treatment could be as high as approximately $1 million per year for a patient weighing 40 kg, as the dose varies by the patient’s age, weight, and insurance coverage.^6,7,8^ Subsequent to eteplirsen’s approval, other treatments in the same class received FDA authorization for DMD based on similarly limited evidence.^9,10,11,12^

Since the FDA-approved indications for novel DMD drugs were not limited to a particular subset of patients with DMD, the population prescribed these drugs in routine clinical care may differ from the patients studied in the drugs’ pivotal clinical trials, raising further questions about the drugs’ efficacy. Thus, we sought to evaluate the demographic and disease characteristics of patients who initiated novel DMD treatments in routine care in different insured populations and compare them with the patients enrolled in the drugs’ pivotal trials. Previous research has found similarities in demographic characteristics and medical costs between patients with DMD and commercial insurance and those with Medicaid coverage, but differences in relation to the costly new medications have not been studied.^13^

## Methods

### Study Design and Data Sources

We conducted a retrospective cross-sectional study using data from the Merative MarketScan (MarketScan) database, Optum’s de-identified Clinformatics Data Mart Database (CDM), and the Transformed Medicaid Statistical Information System Analytic Files. These databases provided comprehensive information on patients from 2 national private insurance networks and the largest US public health insurance provider. The study period was from September 19, 2016 (eteplirsen approval), to December 31, 2018, for Transformed Medicaid Statistical Information System Analytic Files; December 31, 2021, for MarketScan; and March 31, 2022, for CDM (encompassing complete data availability at the time of the study). The Mass General Brigham institutional review board granted approval with a waiver of informed consent to use deidentified claims data. This work is reported based on the Strengthening the Reporting of Observational Studies in Epidemiology (STROBE) reporting guideline.

### Study Cohort

We studied patients initiating novel DMD treatments in routine care settings. The drugs studied included eteplirsen, golodirsen, viltolarsen, and casimersen; they were identified using Healthcare Common Procedure Coding System codes and National Drug Codes for eteplirsen, golodirsen, viltolarsen, and casimersen in outpatient and pharmacy claims (eMethods 1 in Supplement 1). We used a new-user design and assessed patients’ pretreatment characteristics from the time of novel DMD treatment initiation. The cohort entry date was defined as the date of first use of any novel DMD treatment. Patients were required to have 180 days of continuous prior enrollment at baseline, during which they had no novel DMD treatment.

### Baseline Characteristics

Baseline demographic and disease characteristics were measured, including age, sex, race and ethnicity, stage of DMD, and type of novel DMD treatment received on the cohort entry date. Race and ethnicity were patient self-reported in Medicaid and assigned by the data vendor in CDM. Information on race and ethnicity was not available from MarketScan. Race and ethnicity were grouped as White and as racial or ethnic minority (including Asian, Black, Hispanic, and other race and ethnicity) for analysis because of limited sample size for each category individually. We used a validated claims-based staging algorithm to stratify the patients into 4 disease stages, including early ambulatory, late ambulatory, early nonambulatory, and late nonambulatory (eMethods 2 in Supplement 1).^14^ The full list of National Drug Codes used for each marker was derived from previous studies, including oral corticosteroids, such as prednisone and deflazacort, and cardiac medications, such as angiotensin-converting enzyme inhibitors, angiotensin receptor blockers, and β-adrenergic receptor blockers.^14^

In addition, we retrieved summary statistics of baseline demographic and disease-related characteristics of patients receiving novel DMD treatments in the drugs’ pivotal randomized clinical trials using medical and clinical reviews conducted by the FDA Center for Drug Evaluation and Research. These characteristics included age, sex, race and ethnicity, and the stage of DMD progression.^15,16,17,18^ We categorized most patients in the pivotal trials into stage 1 or 2 DMD (if these trials enrolled patients with DMD diagnoses), stable cardiac function, and stable pulmonary function, while excluding those receiving any pharmacologic treatment apart from corticosteroids. We categorized patients in the pivotal trials into stage 3 DMD if they used other cardiac medications. Patients were assumed to be not enrolled in more than 1 pivotal trial as the treatments are indicated for different types of DMD and trigger excision of different exons.

### Outcomes

Patients in the routine care population were followed for 1 year after the date of first use of any novel DMD treatment, with censoring occurring upon discontinuation of the index treatment, disenrollment, death, or reaching the maximum follow-up duration available in a given database. Discontinuation of novel DMD treatment was defined as a gap of more than 1 month during follow-up, with a 30-day exposure grace period. Health care costs were measured using both medical and pharmacy claims, including the patient’s out-of-pocket costs and the payer’s costs, at baseline and during follow-up. The estimated annual baseline costs were calculated as twice the amount of the observed 180-day costs. All costs were converted to 2022 US dollars using the seasonally adjusted Consumer Price Index for all urban consumers.^19^

### Statistical Analysis

Descriptive statistics, including means and SDs, were used to characterize patients in the pivotal trials for all 4 novel DMD treatments. Given the possibility of patients being captured in more than 1 data source (eg, a patient appearing in both CDM and MarketScan), we reported the patient characteristics separately for each database. We used the Fisher exact test to compare categorical characteristics and the *t* test to compare the continuous characteristics between patients in each database and those in the pivotal trials. We assessed the same characteristics between the pivotal trials and the 3 databases among eteplirsen users for sensitivity analysis, as most of the patients who initiated novel DMD treatment were eteplirsen users. We also assessed the discontinuation of novel DMD treatment with a 45-day gap, discontinuation in an early stage (stage 1 and 2) and a later stage (stage 3 and 4) of DMD, and annual health care costs after receiving novel DMD treatments among patients with complete 1-year follow-up data for sensitivity analyses. Data collection was performed using SAS, version 9.4, and analyses were conducted using Stata, version 17 statistical software (StataCorp LLC). A 2-sided *P* < .05 was considered significant.

## Results

### Demographic and Disease Characteristics

Baseline demographic and disease characteristics of patients in the pivotal trials and postapproval clinical settings are presented in Table 1. We identified 58 patients who initiated novel DMD drugs in MarketScan, 35 patients in CDM, and 130 patients in Medicaid. A total of 106 patients receiving novel DMD treatments were identified in the pivotal trials, including 8 eteplirsen users, 25 golodirsen users, 16 viltolarsen users, and 57 casimersen users. The mean (SD) age in the pivotal trials was 8.5 (2.0) years (range, 4.0-13.0 years), which was younger than the mean (SD) age of 13.7 (7.0) years in MarketScan (range, 1.8-33.3 years; *P* < .001), 11.9 (5.7) years in CDM (range, 0.6-23.6 years; *P* < .001), and 13.4 (6.5) years in Medicaid (range, 1.8-46.1 years; *P* < .001). The proportion of female patients identified in postapproval clinical settings was 2.9% (n = 1) in CDM (vs 34 male patients [97.1%]) and 1.5% (n = 2) in Medicaid (vs 128 male patients [98.5%]), which was not different from the pivotal trials. Among the 106 patients in the pivotal trials, 90 (90.6%) were White and 10 (9.4%) were another race or ethnicity, which differed from the racial and ethnic distribution of DMD drug users covered by Medicaid (48 White [76.2%] and 15 others [23.8%]; *P* = .01).

**Table 1.** Demographic and Disease Characteristics of Patients Initiating Novel Duchenne Muscular Dystrophy (DMD) Treatment, 2016-2022

Variable	No. (%)						
	Pivotal trials (n = 106)	MarketScan (n = 58)	*P* value^a^	CDM (n = 35)	*P* value^a^	Medicaid (n = 130)	*P* value^a^
Age, y							
Mean (SD)	8.5 (2.0)	13.7 (7.0)	<.001	11.9 (5.7)	<.001	13.4 (6.5)	<.001
Range	4.0-13.0	1.8-33.3		0.6-23.6		1.8-46.1	
Sex							
Male	106 (100)	58 (100)	NA	34 (97.1)	.25	128 (98.5)	.50
Female	0	0		1 (2.9)		2 (1.5)	
Race and ethnicity^b^							
Racial or ethnic minority group	10 (9.4)	Not available	NA	6/25 (24.0)	.08	15/63 (23.8)	.01
White	96 (90.6)	Not available		19/25 (76.0)		48/63 (76.2)	
Corticosteroid use							
Any	104 (98.1)	32 (55.2)	<.001	16 (45.7)	<.001	84 (64.6)	<.001
Deflazacort	47 (44.3)	19 (32.8)	.19	5 (14.3)	.002	12 (9.2)	<.001
Prednisone	24 (22.6)	13 (22.4)	.99	11 (31.4)	.27	69 (53.1)	<.001
Cardiac medication use	24 (22.6)	23 (39.7)	.02	13 (37.1)	.12	68 (52.3)	<.001
Progression stage of DMD^c^							
1 or 2	103 (97.2)	30 (63.8)	<.001	18 (58.1)	<.001	61 (53.0)	<.001
3	3 (2.8)	10 (21.3)		5 (16.1)		23 (20.0)	
4	0	7 (14.9)		8 (25.8)		31 (27.0)	
Index treatment							
Eteplirsen	8 (7.5)	53 (91.4)	NA	35 (100)	NA	130 (100)	NA
Golodirsen	25 (23.6)	3 (5.2)		0		0	
Viltolarsen	16 (15.1)	2 (3.5)		0		0	
Casimersen	57 (53.8)	0		0		0	

Abbreviations: CDM, Optum’s Clinformatics Data Mart Database; NA, not applicable.

^a^ *P* values are for comparison between the pivotal trial and each claims database, such as pivotal trial vs MarketScan, pivotal trial vs CDM, and pivotal trial vs Medicaid.

^b^ Includes Asian, Black, Hispanic, and other race and ethnicity. In CDM, race was determined using the member’s name and geographic information to derive ethnicity. Members were then mapped to 1 of 4 racial and ethnic categories (Asian, Black, Hispanic, and White) or categorized as unknown or other race and ethnicity. For statistical comparison purposes, we excluded individuals with unknown race and ethnicity from the analysis.

^c^ For statistical comparison purposes, we excluded a minimal number of individuals for whom no progression stage of disease can be categorized from the analysis.

Nearly all patients (103 [97.2%]) in the pivotal trials had an earlier disease stage and were in progression stage 1 or 2 of DMD. By contrast, in the postapproval clinical setting, slightly more than one-third of the patients with DMD were in disease progression stage 3 or 4 when initiating novel DMD treatments (MarketScan, 17 [36.2%; *P* < .001]; CDM, 13 [41.9%; *P* < .001]; Medicaid, 54 [47.0%; *P* < .001]). In sensitivity analyses, we found that all the patients with DMD were in earlier disease stages in the pivotal trial, while less than one-half were in stage 1 or 2 of DMD in Medicaid (61 [46.9%]; *P* = .03) (eTable 1 in Supplement 1).

### Use and Costs of Novel DMD Treatments

The prescribing patterns of novel DMD treatments varied across the databases. Nearly all patients initiated eteplirsen, with 53 patients [91.4%] in MarketScan, 35 (100%) in CDM, and 130 (100%) in Medicaid (Table 1). Golodirsen and viltolarsen were less commonly initiated, with only 3 patients (5.2%) and 2 patients (3.5%) in MarketScan, respectively, initiating these treatments. Medicaid showed an increasing trend over time in the number of patients initiating novel DMD treatments, with 1 patient in 2016 and 91 patients in 2018. In MarketScan, the number of initiators increased from 3 in 2016 to 23 in 2018, decreased to 2 in 2019, and then increased to 15 in 2021. In CDM, no novel DMD initiators were found in 2016 or 2017, and the number of patients initiating novel DMD treatments decreased from 16 in 2018 to 4 in 2022 (Figure).

**Figure.**
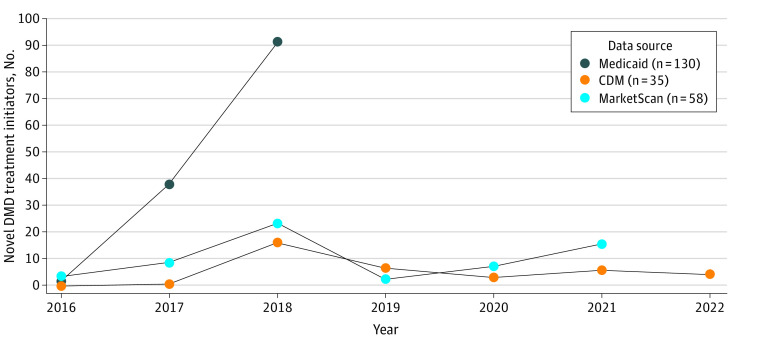
Novel Duchenne Muscular Dystrophy (DMD) Treatment Initiation CDM indicates Optum’s Clinformatics Data Mart Database.

Table 2 presents the annual health care costs for patients with DMD after initiating novel DMD treatments. The payer’s mean (SD) cost for novel DMD treatments varied across the different databases, including $634 764 ($607 101) in MarketScan, $482 749 ($582 350) in CDM, and $384 023 ($1 165 730) in Medicaid. Additionally, approximately one-third of patients discontinued novel DMD treatments after approximately 7 months, with 20 (34.5%) discontinuing over the mean (SD) follow-up period of 6.1 (4.4) months in MarketScan, 13 (37.1%) over 6.9 (3.9) months in CDM, and 39 (30.0%) over 7.2 (4.3) months in Medicaid. Finally, 12 patients (20.7%) in MarketScan, 8 (22.9%) in CDM, and less than 11 (<8.5%) in Medicaid disenrolled from their health insurance after receiving novel DMD treatments (Table 3). In sensitivity analyses, we found a higher treatment discontinuation among patients with later stages (stage 3 and 4) of DMD compared with earlier stages (stage 1 and 2) of DMD in MarketScan and CDM (eTables 2-4 in Supplement 1). We also found higher payer costs for novel DMD treatments in MarketScan (mean [SD], $1 300 246 [$466 298]) and CDM (mean [SD] $1 343 809 [$945 606]) among patients with complete 1-year follow-up data compared with all patients initiating novel DMD (eTable 5 in Supplement 1) and higher payer costs for novel DMD treatments among patients in later DMD progression stages compared with all patients who received novel DMD treatments in Medicaid (mean [SD] $765 097 [$1 824 475]) (eTable 6 in Supplement 1).

**Table 2.** Annual Health Care Costs After Receiving Novel Duchenne Muscular Dystrophy (DMD) Treatments

	MarketScan (n = 58)					CDM (n = 35)				Medicaid (n = 130)			
	Cost, mean (SD), $		Mean difference (95% CI)	*P* value	Cost, mean (SD), $			Mean difference (95% CI)	*P* value	Cost, mean (SD), $		Mean difference (95% CI)	*P* value
	Baseline	Follow-up			Baseline		Follow-up			Baseline	Follow-up		
Total payer’s costs^a^	250 705 (379 115)	685 183 (591 338)	434 477 (260 201 to 608 753)	<.001	420 066 (559 079)		1 076 204 (1 256 655)	656 138 (100 827 to 1 211 449)	.02	425 680 (1 133 154)	431 917 (1 199 704)	3043 (−126 524 to 132 611)	.97
Payer’s costs of novel DMD treatments	NA	634 764 (607 101)			NA		482 749 (582 350)			NA	384 023 (1 165 730)		
Total patient’s OOP costs	3666 (3706)	2710 (3162)	−956 (−2412 to 501)	.19	10 023 (11 981)		8837 (14 447)	−1187 (−5745 to 3372)	.60	NA	NA		
Patient’s OOP costs of novel DMD treatments	NA	1768 (2846)			NA		2618 (3840)			NA	NA		

Abbreviations: CDM, Optum’s Clinformatics Data Mart Database; NA, not available; OOP, out-of-pocket.

^a^ In CDM, the payer’s cost was not directly reported and was estimated as the standard cost reported by CDM minus the total patient’s OOP cost.

**Table 3.** The Follow-Up Status for Patients Receiving Novel Duchenne Muscular Dystrophy Treatments

Variable	MarketScan (n = 58)	CDM (n = 35)	Medicaid (n = 130)
Follow-up time, mean (SD), mo	6.1 (4.4)	6.9 (3.9)	7.2 (4.3)
Reason for end of follow-up, No. (%)			
Complete follow-up (365 d)	14 (24.1)	6 (17.1)	23 (17.7)
Discontinuations of treatment (30-d gap)	20 (34.5)	13 (37.1)	39 (30.0)
Censoring due to disenrollment^a^	12 (20.7)	8 (22.9)	<11 (<8.5)
Administrative censoring due to end of data available^a^	12 (20.7)	8 (22.9)	>57 (>43.8)

Abbreviation: CDM, Optum’s Clinformatics Data Mart Database.

^a^ To avoid violating the Centers for Medicare & Medicaid Services cell size suppression policy, the numbers of participants censored due to disenrollment and administrative censoring due to end of data availability were coarsened.

## Discussion

Novel DMD treatments are intended to slow the progression of muscle weakness and improve quality of life for patients with DMD, although there are no rigorous data on these outcomes from the pivotal trials. In this cross-sectional study, we found that the mean age and disease stage of patients in pivotal trials for novel DMD treatments were substantially lower than seen in routine care. Older patients and patients at later stages of disease may respond differently to these drugs than the relatively younger and healthier patients in the clinical trials, and whether these treatments have any clinical benefit for patients at later stages of DMD remains a question that has not yet been formally tested.^4^

Since novel DMD treatments have been FDA approved based on minimal changes in dystrophin levels seen in muscle biopsy samples—in the case of eteplirsen, with an actual mean (SD) increase to only 0.9% (0.8%) of normal dystrophin levels—manufacturers have promised to conduct confirmatory postapproval trials.^7,20^ For example, a phase 3, multicenter, open-label study of eteplirsen (Study of Eteplirsen in DMD Patients [PROMOVI]) showed similar increases in the surrogate measure end point of dystrophin levels as in the pivotal trial.^5,21^ Although the PROMOVI trial enrolled a larger cohort of patients (n = 79) and included relatively older individuals (mean [SD] age, 9.1 [2.0] years; range, 7-16 years) compared with the pivotal trials, it still excluded patients at more advanced stages of disease who were using medications other than corticosteroids, potentially limiting the assessment of eteplirsen’s benefits for such patients. Other postmarketing confirmatory trials for novel DMD treatments are still incomplete (NCT03992430, NCT02500381, NCT04060199, and NCT02500381) many years after these drugs’ FDA approval.

The high discontinuation rates of novel DMD treatments found in our study merit further investigation. Various factors could contribute to nonpersistence, including lack of efficacy, financial challenges, safety concerns related to side effects, and the presence of other health issues.^8^ Although our study did not assess the specific reasons for discontinuation, the literature suggests the lack of data on any long-term benefits and cardiac function improvements may be a limitation of exon-skipping treatments, which could be the reason for discontinuation.^22,23^

In the MarketScan and Medicaid databases, more than 85% of the payer’s costs for patients with DMD during the observation periods were due to novel DMD treatments. Some payers may perceive an ethical responsibility to ensure that patients with DMD have access to potentially life-improving treatments that are approved by the FDA, but the effectiveness of the exon-skipping treatments for even the population included in pivotal trials is not clear. In such a scenario, high treatment costs incurred by use in a broader population could create financial burdens for payers, potentially limiting their ability to provide other essential health care services. Further study should examine whether the high costs of DMD treatments have led patients to move from private insurance to Medicaid coverage. The financial considerations associated with different DMD treatment options highlight the need for cost-effectiveness evaluations to ensure optimal allocation of resources.

Future studies that aim to enhance the understanding of novel DMD treatments and their postapproval use should take into account several important factors. Incorporating patients with similar prognostic factors as observed in postapproval clinical settings should be a priority in future clinical trials to ensure the relevance of their findings. More data are needed on the actual clinical effectiveness and safety of novel DMD treatments, including in individuals who are older, of minority race and ethnicity, and with more advanced stages of the disease. Additionally, it would be useful to gather more robust routine care setting data, such as disease-specific clinical biomarkers and end points, to supplement the delayed postmarket studies. Access and equity issues, long-term outcomes, and comparative effectiveness across different novel DMD treatments should also be investigated to inform treatment decisions.

### Limitations

Our analysis could not distinguish whether the novel DMD treatments were administered for the specified disease variations they were intended to address, since no information is available on variation subtype in administrative claims data. We expected that the use of novel DMD treatments would adhere to on-label indications, as patients are likely to receive these treatments following genetic testing. Our findings may not be applicable to uninsured patients since our analysis relies on administrative claims data, although most patients using novel DMD treatments are likely to have insurance coverage due to the considerable costs of the drug. Variations in patient age were identified across the 3 insurance claims databases, suggesting that payers may have different coverage policies with regard to who received the drugs. Our analysis may have overestimated discontinuation rates due to the method used to identify treatment discontinuation. Patients who temporarily stopped treatment for more than 1 month but later resumed were not accounted for, although a 30-day grace period should be reasonable as all novel DMD treatments are administered weekly.^15,16,17,18^

Our calculation of average costs included all patients, including those who discontinued and completed the novel DMD treatments during follow-up. This approach may have led to an underestimation of costs, particularly for patients in later stages of the disease who incurred higher treatment expenses.^24^ In our sensitivity analyses focusing on all patients with a full year of follow-up, we observed higher annual payer costs for novel DMD treatments compared with our main study results, except for patients within the Medicaid program, most of whom were in capitated payment plans. The Medicaid and CHIP Payment and Access Commission has reported that the substantial costs associated with novel advanced therapies pose challenges in terms of management and introduce budget volatility that Medicaid managed care plans with annual capitated contracts may not easily accommodate.^25^

Limitations in our data source include potential overlap of patients between the CDM and MarketScan databases, preventing us from combining the data for a unified analysis. The accuracy of race and ethnicity information in the CDM may have been low, since CDM uses a proprietary algorithm to assign race and ethnicity derived using member geographic location and name.^26^ Furthermore, the categorization of progression stages in the pivotal trials did not align with our methodology for patients in the claims databases.^14^ We made the conservative assumption that patients who used cardiac medications were in the early nonambulatory phase of DMD, which may underestimate the true difference between patients in clinical trials and routine practices. Discrepancies in progression stage categorization may be associated with the distribution of stages when comparing the 2 data sets, introducing potential bias and limiting direct comparisons.

Finally, we found minimal use of golodirsen and viltolarsen and no use of casimersen. Although we anticipated similar patient characteristics in the routine care setting across these novel DMD treatments, given that their only distinction lies in their targeting of different disease variations, future studies should investigate and compare patient profiles specific for these drugs.

## Conclusions

In this cohort study, we reviewed the characteristics, adherence, and economic implications of novel DMD treatments in routine care settings. The differences observed between patients in pivotal trials and those in routine care settings raise important considerations regarding the effectiveness of these treatments in diverse patient populations. In particular, the minimal clinical benefit claimed in the preapproval studies of these drugs could be even smaller in routine use. The high discontinuation rates, economic burden, and access challenges associated with novel DMD treatments highlight the need for further research and interventions to rationalize their use.

## Notes

Supplement 1. **eMethods 1.** HCPCS and NDCs of Novel DMD Treatments **eMethods 2.** Clinical Definition of Progression Stages of DMD **eTable 1.** Demographic and Disease Characteristics of Patients Initiating Eteplirsen: 2016-2022 **eTable 2.** The Follow-Up Status for Patients Receiving Novel DMD Treatments (45-Day Gap) **eTable 3.** The Follow-Up Status for Patients Receiving Novel DMD Treatments With Stage 1 or 2 DMD **eTable 4.** The Follow-Up Status for Patients Receiving Novel DMD Treatments With Stage 3 or 4 DMD **eTable 5.** Annual Health Care Costs After Receiving Novel DMD Treatments Among Patients With Complete 1-Year Follow-Up **eTable 6.** Annual Health Care Costs After Receiving Novel DMD Treatments Among Patients With Stage 3 or 4 DMD

Supplement 2.
Data Sharing Statement
