# Supplementary material for: Characteristics of Patients Receiving Novel Muscular Dystrophy Drugs in Trials vs Routine Care
Source: JAMA Netw Open. 2024 Jan 24;7(1):e2353094. doi: 10.1001/jamanetworkopen.2023.53094 (PMC10809016; doi:10.1001/jamanetworkopen.2023.53094)
Supplement: Supplement 1. — eMethods 1. HCPCS and NDCs of Novel DMD Treatments eMethods 2. Clinical Definition of Progression Stages of DMD eTable 1. Demographic and Disease Characteristics of Patients Initiating Eteplirsen: 2016-2022 eTable 2. The Follow-Up Status for Patients Receiving Novel DMD Treatments (45-Day Gap) eTable 3. The Follow-Up Status for Patients Receiving Novel DMD Treatments With Stage 1 or 2 DMD eTable 4. The Follow-Up Status for Patients Receiving Novel DMD Treatments With Stage 3 or 4 DMD eTable 5. Annual Health Care Costs After Receiving Novel DMD Treatments Among Patients With Complete 1-Year Follow-Up eTable 6. Annual Health Care Costs After Receiving Novel DMD Treatments Among Patients With Stage 3 or 4 DMD [file jamanetwopen-e2353094-s001.pdf]

Supplemental Online Content

Hong D, Avorn J, Wyss R, Kesselheim AS. Characteristics of patients receiving novel muscular dystrophy drugs in trials vs routine care. *JAMA Netw Open*. 2024;7(1):e2353094. doi:10.1001/jamanetworkopen.2023.53094

eMethods 1. HCPCS and NDCs of Novel DMD Treatments

eMethods 2. Clinical Definition of Progression Stages of DMD

eTable 1. Demographic and Disease Characteristics of Patients Initiating Eteplirsen: 2016-2022

eTable 2. The Follow-Up Status for Patients Receiving Novel DMD Treatments (45-Day Gap)

eTable 3. The Follow-Up Status for Patients Receiving Novel DMD Treatments With Stage 1 or 2 DMD

eTable 4. The Follow-Up Status for Patients Receiving Novel DMD Treatments With Stage 3 or 4 DMD

eTable 5. Annual Health Care Costs After Receiving Novel DMD Treatments Among Patients With Complete 1-Year Follow-Up

eTable 6. Annual Health Care Costs After Receiving Novel DMD Treatments Among Patients With Stage 3 or 4 DMD

This supplemental material has been provided by the authors to give readers additional information about their work.

© 2024 Hong D et al. *JAMA Network Open.*

eMethods 1. HCPCS and NDCs of Novel DMD Treatments

Generic name	HCPCS	NDC
eteplirsen	J1428	60923028410
golodirsen	J1429	60923046502
viltolarsen	J1427	73292001101
casimersen	J1426	60923022702

eMethods 2. Clinical Definition of Progression Stages of DMD

	Criteria 1	Criteria 2
Stage 1 (Early ambulatory)	Able to perform 10MWR	Able to perform RFS
Stage 2 (Late ambulatory)	Able to perform 10MWR	Unable to perform RFS
Stage 3 (Early non-ambulatory)	Unable to perform 10MWR	FVC%p > 50%
Stage 4 (Late non-ambulatory)	Unable to perform 10MWR	FVC%p ≤ 50% (Confirmed by two consecutive visits)

*10MWR 10 m walk run test, RFS rise from supine, FVC%p forced vital capacity percentage predicted (values >80% are considered to be normal.) The progression markers and the related validated claims-based staging algorithm, including International Classification of Diseases, Ninth/ Tenth Revision (ICD-9/10), Healthcare Common Procedure Coding System (HCPCS), Current Procedural Terminology (CPT) and National Drug Code (NDC) codes, can be found in the previous study.[14]

© 2024 Hong D et al. *JAMA Network Open.*

eTable 1. Demographic and Disease Characteristics of Patients Initiating Eteplirsen: 2016-2022

Variables	Pivotal Trial (n=8)	MarketScan (n=53)	P-value	CDM (n=35)	P-value	Medicaid (n=130)	P-value
Age, mean years (SD)	8.9 (0.99)	14.0 (7.1)	0.05	11.9 (5.7)	0.15	13.4 (6.5)	0.05
Age range, years	7-10	1.8 – 33.3		0.6 – 23.6		1.8 – 46.1	
Sex, n (%)							
Male	8 (100)	53 (100)	NA	34 (97.1)	1.00	128 (98.5)	1.00
Female	0 (0)	0		1 (2.9)		2 (1.5)	
Race/Ethnicity, n (%) [a]							
Non-White	1 (12.5)	NA		6 (24.0)	0.65	15 (23.8)	0.67
White	7 (87.5)	NA		19 (76.0)		48 (76.2)	
Steroid use, n (%)							
Any steroid use	8 (100)	27 (50.9)	0.016	16 (45.7)	0.006	84 (64.6)	0.051
Deflazacort	NR	15 (28.3)	NA	5 (14.3)	NA	12 (9.2)	NA
Prednisone	NR	12 (22.6)	NA	11 (31.4)	NA	69 (53.1)	NA
Cardiac medication use, n (%)	0	19 (35.94)	NA	13 (37.1)	NA	68 (52.3)	NA
Progression Stage of DMD, n (%) [b]							
Stage 1 or 2	8 (100)	28 (52.8)	0.22	18 (51.4)	0.14	61 (46.9)	0.03
Stage 3	0	9 (17.0)		5 (14.3)		23 (17.7)	
Stage 4	0	6 (11.3)		8 (22.9)		31 (23.9)	

Note: [a] Non-White include Black, Asian, Hispanic, and Other race. In the CDM database, race is determined using the member's name and geographical information to derive ethnicity. Based on this derived ethnicity, members are then mapped to one of five race categories (Asian, Black, Hispanic, and White) or categorized as Unknown/Other. For statistical comparison purposes, we excluded individuals with Unknown race/ethnicity from the analysis; NA: not available. P-values are for comparison between the pivotal trial and each claims database, such as pivotal trial vs. MarketScan, pivotal trial vs. CDM, and pivotal trial vs. Medicaid. [b] For statistical comparison purposes, we excluded a minimum number of individuals with no progression stage of DMD can be categorized from the analysis.

eTable 2. The Follow-Up Status for Patients Receiving Novel DMD Treatments (45-Day Gap)

Variables	MarketScan (n=58)	CDM (n=35)	Medicaid (n=130)
Mean follow-up time, months (SD)	6.3 (4.3)	7.3 (3.8)	7.5 (4.1)
Reason for end of follow-up, n (%)			
Complete follow-up (365 days)	15 (25.9)	9 (25.7)	27 (20.8)
Discontinuations of treatment (45-day gap)	19 (32.7)	10 (28.5)	35 (26.9)
Censoring due to disenrollment	12 (20.7)	8 (22.9)	<11 (<8.5)
Administrative censoring due to end of data available	12 (20.7)	8 (22.9)	>57 (43.8)

eTable 3. The Follow-Up Status for Patients Receiving Novel DMD Treatments With Stage 1 or 2 DMD

Variables	MarketScan (n=30)	CDM (n=18)	Medicaid (n=61)
Mean follow-up time, months (SD)	6.9 (4.5)	8.3 (3.6)	7.3 (4.3)
Reason for end of follow-up, n (%)			
Complete follow-up (365 days)	10 (33.3)	4 (22.2)	14 (22.9)
Discontinuations of treatment	7 (23.3)	5 (27.8)	19 (31.1)
Censoring due to disenrollment	5 (16.7)	7 (38.9)	3 (4.9)
Administrative censoring due to end of data available	8 (26.7)	2 (11.1)	25 (40.1)

eTable 4. The Follow-Up Status for Patients Receiving Novel DMD Treatments With Stage 3 or 4 DMD

Variables	MarketScan (n=17)	CDM (n=13)	Medicaid (n=54)
Mean follow-up time, months (SD)	4.9 (4.2)	5.3 (4.0)	7.2 (4.2)
Reason for end of follow-up, n (%)			
Complete follow-up (365 days)	2 (11.7)	2 (15.4)	8 (14.8)
Discontinuations of treatment	8 (47.1)	6 (46.1)	15 (27.8)
Censoring due to disenrollment	4 (23.5)	1 (7.7)	1 (1.8)
Administrative censoring due to end of data available	3 (17.7)	4 (30.8)	30 (55.6)

© 2024 Hong D et al. *JAMA Network Open.*

eTable 5. Annual Health Care Costs After Receiving Novel DMD Treatments Among Patients With Complete 1-Year Follow-Up

	MarketScan (n=14)				CDM (n=6)			
Mean Annual healthcare costs, $ (SD)	Baseline	Follow-up	Mean Difference (95% CI)	P-value	Baseline	Follow-up	Mean Difference (95% CI)	P-value
Total payer's costs *	208,383 (362797.0)	1,335,685 (451,258)	1,127,302 (769,565, 1,485,038)	<0.001	343,641 (586,871)	2,922,491 (2,178,916)	2,578,851 (- 1,428,615 6,586,316)	0.133
Payer's costs of novel DMD treatments	NA	1,300,246 (466,298)			NA	1,343,809 (945,606)		
Total patient's OOP costs	1,934 (1,893)	5,138 (3,574)	3,204 (1,144, 5,264)	0.005	21,607 (22685)	32,894 (26352)	11,287 (16,625 39,199)	0.288
Patient's OOP costs of novel DMD treatments	NA	3,682 (3,727)			NA	7,354 (5,688)		

Note: * In CDM, payer's cost was not directly reported, and was estimated as the standard cost amount reported by CDM – patient's OOP costs. We did not include Medicaid patients in this sensitivity analysis because 91% of the patients with Medicaid (n=21) with 1-year complete follow up were enrolled in managed care plans with capitated payments. OOP: out-of-pocket. CI: Confidence Interval.

© 2024 Hong D et al. *JAMA Network Open.*

eTable 6. Annual Health Care Costs After Receiving Novel DMD Treatments Among Patients With Stage 3 or 4 DMD

	MarketScan (n=17)				CDM (n=13)				Medicaid (n=54)			
Mean Annual healthcare costs, $ (SD)	Baseline	Follow-up	Mean Difference (95% CI)	P-value	Baseline	Follow-up	Mean Difference (95% CI)	P-value	Baseline	Follow-up	Mean Difference (95% CI)	P-value
Total payer's costs *	289,921 (433,310)	618,909 (597,189)	328,923 (49,747, 608,099)	0.02	421,668 (645,181)	1,042,636 (1,907,458)	620,967 (-1,021,198, 2,263,132)	0.41	654,313 (1,616,618)	771,705 (1,771,452)	83,826 (-199,979, 367,630)	0.55
Payer's costs of novel DMD treatments	NA	571,318 (592,714)				458,821 (843,783)				765,097 (1,824,475)		
Total patient's OOP costs	4,396 (3,256)	2,738 (2,748)	-1,658 (-4,110, 794)	0.17	18,222 (14,905)	13,272 (23,294)	-4,951 (-15,751, 5,849)	0.32	NA	NA		
Patient's OOP costs of novel DMD treatments	NA	1,646 (2,051)				2,250 (4,047)			NA	NA		

Note: * In CDM, the payer cost was not directly reported, and was estimated as the standard cost amount reported by CDM minus patient's OOP costs. OOP: out-of-pocket. CI: Confidence Interval.

© 2024 Hong D et al. *JAMA Network Open.*
