# Supplementary material for: Characteristics of Patients Receiving Novel Muscular Dystrophy Drugs in Trials vs Routine Care
Source: JAMA Netw Open. 2024 Jan 24;7(1):e2353094. doi: 10.1001/jamanetworkopen.2023.53094 (PMC10809016; doi:10.1001/jamanetworkopen.2023.53094)
Supplement: Supplement 2. — Data Sharing Statement [file jamanetwopen-e2353094-s002.pdf]

Data Sharing Statement

Hong. Characteristics of Patients Receiving Novel Muscular Dystrophy Drugs in Trials vs Routine Care. *JAMA Netw Open*. Published January 24, 2024. doi:10.1001/jamanetworkopen.2023.53094

Data
Data available: No

Additional Information
Explanation for why data not available: Primary data available to investigators under DUA that does not permit public posting
